# Montreal Veterinary Medical Association

**Published:** 1897-12

**Authors:** W. B. Wallis

**Affiliations:** Secretary


Secretary.

### MONTREAL VETERINARY MEDICAL ASSOCIATION.

THE regular meeting was held on Thursday evening, October 20th, in the library, the first Vice-President, Prof. Baker, occupying the chair.

There were present also Dr. Duncan McEachran, Dr. Sugden, and a full attendance of members.

After roll-call and the reading of minutes, it was unanimously voted that the Secretary should order Georg Müller's *Diseases of the Dog*, and P. J. Cadiot's *Treatise on Veterinary Surgical Therapeutics of the Domestic Animals*, as additions to the library of the Society.

The President then called on Mr. Lambert for his case-report, which proved to be one of "Impaction of the Colon in a Mule," rendered interesting from the fact that he had obtained no results from the use of such powerful drugs as croton oil and eserine, and that the post-mortem showed the colon to be decidedly subnormal in size. Mr. Lambert explained his reasons for resorting to the above treatment, by stating that, as is so often the case, he was not called in professionally until the owner had already reduced the patient to a hopeless condition by the ridiculous use of numerous quack-remedies. A discussion ensued, assisted by the Honorary President and President as to the causes, symptoms, and treatment of impaction, and as a result the prevailing opinion was that the wisest course to pursue was one of patience and perseverance in dilating the intestinal contents with a plentiful supply of mucilaginous drenches and rectal enemas in preference to the use of dangerous drugs, which were only occasionally successful.

Mr. Spanton then followed with an interesting essay on the subject of "Purpura Hemorrhagica," in which he showed how, from the earliest times of veterinary writings, the irregularity of the symptoms, etiological obscurity and different degrees of severity of this disease had caused a varied nomenclature, stating that the one positive symptom in mild cases, otherwise difficult of diagnosis, was the appearance of purple spots on the Schneiderian membrane. After carefully describing the symptoms, he stated that the idiopathic swellings were due to the congested condition of the arterioles and capillaries, owing to vasomotor paralysis, pointing out the danger of asphyxia should extravasation of blood occur in the lungs. Amongst the many conditions which are supposed to be the causes of this disease, he gave special importance to that of improper hygienic surroundings, quoting Prof. Williams as his authority for saying that "Purpura was due to the absorption of products of decomposition extrinsic to the body, and that animals not fully recovered from some former debilitating disease were predisposed to it. He mentioned the difficulty in forming a prognosis, as frequently a patient to all appearances making a satisfactory and rapid recovery might at the next visit be found at death's door. The post-mortem symptoms were then fully described, and in conclusion he mentioned the various modes of treatment, recommending as preventive measures a stimulating course for patients suffering from diseases of the respiratory organs, such as influenza, accompanied as they were by a low type of fever. A long discussion ensued, in which the advisability of scarifying or fomenting the swellings was argued, it being generally concluded that the former course was unwise, whereas the latter, according to the experience of some, had frequently been of the greatest benefit. Dr. Duncan McEachran then pointed out what a large field this disease presented for elucidation. In his opinion the thickening of the bronchial mucous membrane, caused by an attack of influenza, prevented the air from properly reaching the blood, and as a consequence of this impoverished condition of the blood a relaxed condition of the walls of the smaller vessels was produced, permitting the transudation of the fluid into the cellular tissue at the most dependent parts of the body. He considered scarifying injudicious, inasmuch as the natural healing power was already very weak, and there was a great tendency for it to go on to gangrene, therefore every caution should be taken to prevent an abrasion. He fully indorsed the recognized treatment of chlorate of potash or turpentine, advising also the persistent use of hot fomentations. The chairman stated there was no disease like it in peculiarity, mentioning the shifting and sudden extent of the swellings and the apparently normal condition of the patient otherwise, as indicated by the pulse, temperature, and eyes. This disease, he said, should stimulate pathologists, and, as regards the treatment with an intratracheal injection of a solution of iodine, of which he had only one unsuccessful experience, he recommended this to the consideration of the experimental committee. He then held over the discussion on this subject, and, there being no further business, adjourned the meeting.

The regular meeting was held on the 11th ult., in the library, the President, Prof. Charles McEachran, occupying the chair. There were also present Drs. Martin, Thurston, and Sugden, and a full attendance of members. After roll-call and the reading of the minutes the question was raised as to the binding in yearly volumes of the various magazines belonging to the Society, and the President appointed Messrs. Spanton and Wallis to look them over and report any numbers that might be missing, in order that they should be replaced.

Mr. Pfersick then reported an operation for the removal of a fourth upper molar in a horse, by trephining. He stated that the patient had been treated by a quack for three months for indigestion, and, no improvement being made, the owner had come to him. On examination he found the animal to be suffering from a decayed upper molar on the right side, and that there was only a slight extension projecting above the gum An attempt to seize this with forceps failed, owing to the stump breaking off; and, the owner insisting upon having it removed, Mr. Pfersick decided to trephine. This operation he performed successfully after placing the animal under the influence of chloroform, treating the resulting wound with daily administrations of antiseptic plugs and injections. Two months later the owner informed him that the cavity was healing up rapidly, and the general condition of the animal had improved greatly. A short discussion ensued as to the advisability of undertaking this operation frequently, the chairman stating that out of many experiences of his own but few had done well, owing partly to the neglect of attendants in keeping the socket clean and to the trouble caused by the opposite molar growing up into the vacant space. He requested that the case be again reported on.

Mr. Paquin followed with an essay on "Rabies." After tracing its history from the time of Aristotle to the present, he enumerated the various theories as to its causes, the most direct one being that of inoculation with the saliva of a rabid animal. The different periods of incubation, which he stated might extend from three days to a year or more, were attributed to the quantity of the virus inoculated, also the extent and locality of the wound. The symptoms of the raving form and the mute or paralytic form were carefully described, as was the manner in which the animal, being at one stage unable to swallow, would go into convulsions on being given water, which had been the cause of this disease being erroneously called hydrophobia. As regards differential diagnosis between this and certain other diseases with which it might be confounded, he especially pointed out the inclination of an animal to bite itself or any object coming across its path, and to swallow, before paralysis of the pharynx occurred, stones, chips, and other foreign matter. As preventive measures after inoculation, he stated that cauterization or excision of the wound, if performed within a reasonable time, had in most cases enabled people to escape, owing to the fortunate fact that the virus was very slowly absorbed. The wisest course to pursue, however, was at once to go to the nearest Pasteur Institute, where a cure and immunity for from three to five years could be obtained in 99 per cent. of the cases. A vapor-bath at 120° for fifteen or twenty minutes was also often successful. This cure was discovered by Dr.

Buisson thirty-five years ago, who, having been bitten by a rabid patient, and the symptoms being developed in himself, resolved to die in a vapor-bath, which, to his surprise, completely cured him. In conclusion, he pointed out the folly of killing dogs that had bitten people, when the proper course should be to keep them quietly in confinement to notice whether symptoms of rabies were developed or not.

Dr. Martin, in response to a request from the chair, then addressed the meeting. He stated that rabies attracted more attention in Europe, owing to its greater prevalence there. During a visit to Paris he had spent some time at the Pasteur Institute, and had been much struck with the affectionate manner of the afflicted animals; also their peculiar shrieking bark, which was so characteristic that people were able to detect a rabid animal by hearing it. It had been proved, he said, that the virus travelled along the nervous system, for if a subject were inoculated, and the nerve trunk then divided, it would often escape. Continuing, he described the method in which the virus was prepared for inoculation against the disease, and, with regard to the periods of incubation, stated that a bite on the face was most rapidly fatal.

The President then thanked Dr. Martin and congratulated the Society upon the opportunity they had had of listening to such an able authority. Considering the ground had been so well covered, he would not detain them beyond mentioning that it was not necessary to have a scar; the mere fact of having one's face licked by a rabid dog was sufficient for inoculation. The disease, he said, was most frequently seen in foxhounds and mongrels, owing to their habits of cohabitation, but during his practice in Canada he had never yet come across a case, though it was stated that an officer in Quebec some years ago had died from the bite of a rabid fox.

There being no further business the meeting was adjourned.

W. B. WALLIS,
Secretary.

